# A Benford’s law-based framework to determine the threshold of occurrence sites for species distribution modelling from ecological monitoring databases

**DOI:** 10.1038/s41598-023-44010-z

**Published:** 2023-10-05

**Authors:** Taeyong Shim, Zhonghyun Kim, Jinho Jung

**Affiliations:** 1 Ojeong Resilience Institute, Korea University, Seoul, 02841 Republic of Korea; 2 Institute of Life Science and Natural Resources, Korea University, Seoul, 02841 Republic of Korea; 3 Division of Environmental Science and Ecological Engineering, Korea University, Seoul, 02841 Republic of Korea

**Keywords:** Scientific data, Ecology

## Abstract

The use of data-based species distribution models (SDMs) has increased significantly in recent years. However, studies of determining the minimum requirements of occurrence sites from ecological monitoring datasets used in species distribution modelling remain insufficient. Therefore, this study proposed a framework to determine the threshold of minimum occurrence sites for SDMs by assessing compliance with Benford’s law. The compliance test verified that the national-scale freshwater fish monitoring dataset was natural and reliable. Results derived from true skill statistics (TSS) determined the minimum number of occurrence sites for reliable species distribution modelling was 20 with a TSS value of 0.793 and an overall accuracy of 0.804. The Benford compliance test has shown to be a useful tool for swift and efficient evaluation of the reliability of species occurrence datasets, or the determination of the threshold of occurrence sites before species distribution modelling. Further studies regarding the evaluation of this method’s transferability to other species and validation using SDM performance are required. Overall, the framework proposed in this study demonstrates that Benford compliance test applied to species monitoring datasets can be used to derive a universal and model-independent minimum occurrence threshold for SDMs.

## Introduction

Species distribution models (SDMs) generate relationships between abiotic and biotic factors and species occurrence records to predict the probability of species presence^1–3^. Over the last several decades, SDMs have been widely used in various applications^1,4,5^, including species conservation^6^, climate change impact assessment^7,8^, invasive species management^9,10^, and paleoecology^5^.

SDM users generally collate species occurrence data from ecological monitoring datasets that pass quality assurance and quality control procedures conducted during the dataset construction phase^11,12^. These procedures usually focus on defining rules to ensure the integrity of the dataset^13^ or detecting and correcting errors within the dataset^12,14^. In addition, sufficient occurrence sites (or sample sizes) are recommended for SDMs, since the model performance deteriorates when the number of occurrence sites is too low^15–18^. Previous studies have attempted to determine the minimum number of occurrence sites (e.g., 5 sites to 200 sites) by evaluating model accuracy (e.g., Pearson’s r, area under the receiver operating characteristic curve, weighted kappa, etc.)^3,15,17–19^. However, these thresholds are specific and model-dependent^19,20^, and should be verified using independent data^21,22^. Thus, a more generalized procedure is required to determine the reliability of a dataset and the minimum amount of occurrence data for SDM applications.

**Figure 1:**
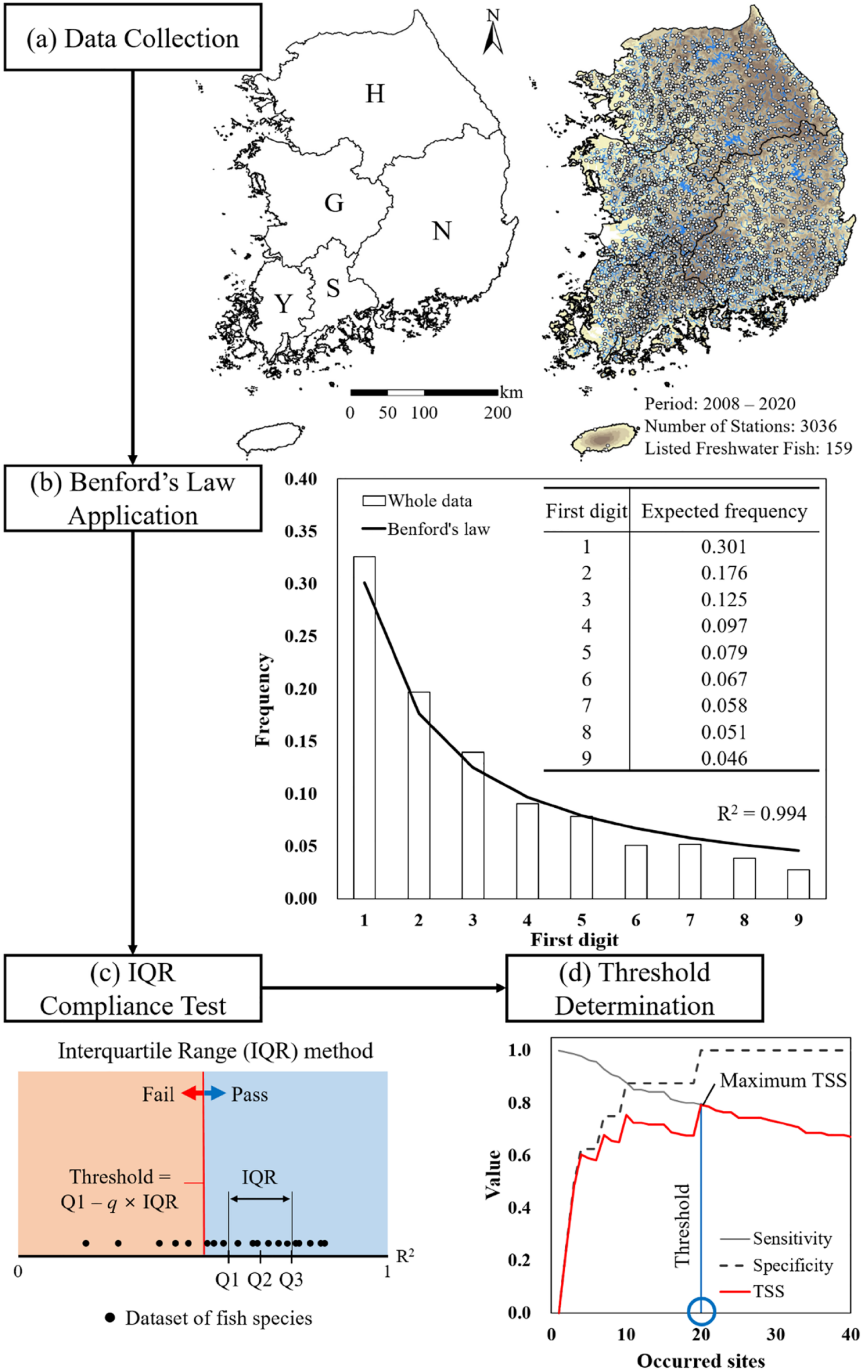
Methodologies used in this study: (**a**) Monitoring data of freshwater fish (details are available in Table S1) in South Korea were collected (Han, Nakdong, Geum, Yeongsan, and Seomjin river watersheds, represented by their first letter; H, N, G, Y, and S, respectively); (**b**) Compliance of whole fish dataset (white bars) with Benford’s law (solid line) evaluated by the coefficient of determination (R^2^); (**c**) Compliance of single fish dataset with Benford’s law evaluated by the interquartile range (IQR) method; and (**d**) Determination of the minimum number (threshold) of occurrence sites (blue circle) using true skill statistics (TSS). The maps were generated using ArcGIS Pro (ESRI, ver. 3.1; https://www.esri.com/en-us/arcgis/products/arcgis-pro/overview).

Hence, this study aimed to develop a novel methodology using Benford’s law as a universal and model-independent criterion to identify the minimum number of occurrence sites required for SDMs from species occurrence datasets. As a case study, the reliability of a national freshwater fish monitoring dataset, which was collected for 13 consecutive years throughout South Korea, was evaluated (Fig. 1a).

### Benford’s law

Benford’s law was discovered by Simon Newcomb^23^ and justified by Frank Benford^24^; it refers to a phenomenon in which the distribution of leading (non-zero) digits from a dataset that occurs naturally (or untampered) complies with a logarithm equation (Eq. 1)^25,26^:

1 $$\mathrm{P}({l}_{i})={\mathrm{log}}_{10}(1+\frac{1}{{l}_{i}}), {l}_{i}\in \{1, 2, 3, 4, 5, 6, 7, 8, 9\}$$

where P is the expected frequency (or probability) of the first digit (*l*_*i*_). The expected frequency for each digit is presented in Fig. 1b.

In practice, Benford’s law is frequently applied as a standard when evaluating digit distributions. It is assumed that the dataset will not comply with Benford’s law if the numbers are not natural and influenced by human choice^26^. Compliance with Benford’s law is determined through goodness-of-fit tests by comparing the frequency of digits that appear in the dataset^27^. The most popular and widespread use of this law is in fraud detection, including data fabrication and falsification^25,28,29^. In the field of environmental science, Benford’s law has been applied to secure the reliability or identify anomalies in datasets, which include stream flows^27^, earthquakes^30^, tropical cyclones^31^, ecosystem naturalness^32^, health/disease report ^33^, ecotoxicity^26^, and phytoplankton cells in colonies^34^ and abundance^35^.

## Results and discussions

### Evaluating the reliability of the fish monitoring dataset

In this study, Benford’s law was applied to evaluate the reliability of the entire fish monitoring dataset (Table S1). The frequency of the first digits extracted from the total dataset complied well with Benford’s law, yielding a coefficient of determination (R^2^) of 0.994 (Fig. 1b). Considering that *R*^2^ > 0.85 is generally accepted as high credibility^36,37^, it confirms that the national freshwater fish monitoring dataset is highly reliable. In general, compliance with Benford’s law indicates that the dataset is authentic and natural^26,27,31^. Thus, the national freshwater fish monitoring dataset sufficiently represents the occurrence of freshwater fish in South Korea.

Datasets that do not comply with Benford’s law generally result from insufficient data quantity (or incomplete datasets), excessive rounding of data, and data errors^27^. For instance, Polidori and Hage^38^ applied Benford’s law to evaluate the accuracy of elevation, slope, and stream order from a digital elevation model and found large errors in elevation. Moreover, Noleto–Filho et al.^39^ demonstrated that the compliance assessment of a Brazilian fishing dataset with Benford’s law could identify the cause of unreliability. These findings suggest that Benford’s law can be used as a solid criterion for evaluating the reliability of monitoring datasets^40^.

### Determining the threshold of fish occurrence sites

**Figure 2:**
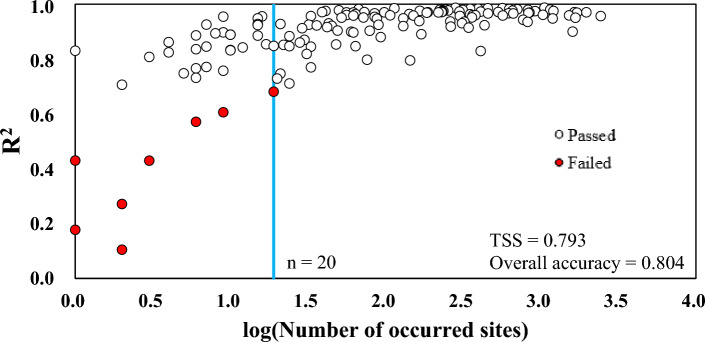
Determination of the minimum fish occurrence site threshold (blue line) using TSS. Compliance (viz., passed or failed) of the single fish dataset with Benford’s law, evaluated by the IQR method, is denoted in white and red circles, respectively. The maximum TSS value (0.793), with an overall accuracy of 0.804, was yielded when the minimum number was set at 20 sites.

Benford’s law was also applied to determine the minimum number of fish occurrence sites required for species distribution modelling (Table S2). The interquartile range (IQR) method (Fig. 1c) showed that the species with *R*^2^ < 0.698 did not comply with Benford’s law in which 8 species failed among the 148 species tested (Fig. 2). Moreover, true skill statistics (TSS) was used to determine the threshold of minimum occurrence sites for complying with Benford’s law (Fig. 1d), since a reliable threshold can be produced by maximizing the sum of sensitivity and specificity (equivalent to maximizing the TSS value) than other methods (e.g., maximizing overall accuracy, maximizing kappa value, using the mean predicted value, etc.)^41^. The maximum TSS value of 0.793 was derived at 20 occurrence sites, with an overall accuracy of 0.804 (Fig. 2). Since TSS surpassed the criterion of 0.6^42,43^, the threshold was shown to effectively discriminate compliance with Benford’s law for each fish species according to the number of occurrence sites. As suggested in Szabo et al.^40^, one of the advantages of Benford’s law is the efficient and swift evaluation process, since modelling each species individually with SDMs entails a higher cost. These findings suggest that Benford’s law can be used as a universal tool for determining the minimum number of fish occurrence sites since it is independent of SDMs.

As demonstrated in this study, Benford’s law can be utilized to evaluate the reliability of species monitoring datasets. However, the results obtained from the compliance test provide limited ecological information^40^. For instance, a species monitoring dataset can be determined to be reliable, but information on species richness or biodiversity is not identified. Meanwhile, failing the compliance test implies additional or detailed investigation is required to ensure the reliability of using the dataset. Thus, Benford compliance test can be used as a screening process to evaluate the reliability of species monitoring or larger ecological datasets^40^, or to determine the threshold of occurrence sites before developing SDMs.

Further studies of applying this approach to SDMs and validating the threshold of species occurrence with model accuracy are required. Concurrently, datasets from various regions and other groups of organisms (e.g., amphibians, avians, invertebrates, etc.) should be tested for transferability of the approach, because the freshwater fish dataset was the only available dataset with detailed monitoring records that were collectable for this study.

## Conclusions

This study demonstrated that Benford compliance test based on species occurrence datasets can provide a universal and model-independent criterion for determining the minimum occurrence threshold for species distribution modelling. A national-scale freshwater fish monitoring dataset was verified to comply with Benford’s law, indicating that the fish monitoring dataset was reliable and natural. Through the TSS, 20 was determined as the minimum occurrence threshold for modelling the distribution of freshwater fish from this dataset. Further studies of testing species occurrence datasets of other groups of organisms or regions are required to verify the transferability of this method. Also, future studies should evaluate the performance of this approach by comparing SDM accuracy divided by the threshold of species occurrence.

## Methods

### Data collection

The freshwater fish ecological monitoring data from 2008 to 2020 were collected from the Water Environment Information System (https://water.nier.go.kr; initially accessed on June 05, 2017, and updated on August 03, 2021). Along with the species occurrence results, the attributes regarding the monitoring program were included in the dataset. In addition, all survey stations were assigned to one of the 5 basins (Han, Nakdong, Geum, Seomjin, and Yeongsan River Basins) according to the classification available in WAMIS (https://wamisgo.kr accessed on August 03, 2021.). Details of the collected data and their statistics are listed in Table S1. Among the 159 fish species initially listed, 11 were excluded from the analysis due to non-occurrence. Data archiving and statistical analysis were conducted using MS Excel 2019 (Microsoft Corporation, Redmond, WA, USA).

### Evaluating compliance with Benford’s law for species occurrence dataset

Benford’s law was applied by extracting the leading (or first) digit from the collected dataset. Subsequently, the frequency (0–9) of each digit (1–9) was calculated followed by a compliance test. The whole dataset used the entire dataset (W in Table S1), while each fish species used a species-specific dataset, respectively (S in Table S1). The coefficient of determination (R^2^) was derived by the regression analysis of the first digit frequency from the species monitoring dataset (i.e., whole dataset, datasets of each fish species) and Benford’s law (Fig. 1b). In addition, the occurrence data of freshwater fish in the 5 basins was integrated in the compliance tests assuming that SDM users generally use the largest range of available occurrence data^44^.

Compliance with Benford’s law was determined using the IQR (interquartile range) method (Fig. 1c). The R^2^ was classified into pass (*R*^2^ ≥ threshold of compliance) and fail (*R*^2^ < threshold of compliance) according to the following equations (Fig. 1c; Eqs. 2, 3):

2 $$\mathrm{IQR }=\mathrm{ Q}3 -\mathrm{ Q}1$$

3 $$\mathrm{Threshold of Compliance }=\mathrm{ Q}1 - q\times \mathrm{IQR}$$

where Q3 is the upper 25% quartile of R^2^s and Q1 is the lower 25% quartile of R^2^s acquired from the regression analysis of each species. Meanwhile, *q* is the coefficient that determines the threshold location, where a common value of 1.5 was applied in this study^45,46^.

### Determining minimum occurrence site threshold

The required minimum occurrence sites for species distribution modelling was determined using true skill statistics (TSS) according to Allouche et al.^47^. TSS was conducted using a 2 × 2 contingency table (Table S3), where “a” is the number of species that accurately passed the threshold, “b” is the number of species that incorrectly passed the threshold (type I error; false positive), “c” is the number of species that incorrectly did not pass the threshold (type II error; false negative), and “d” is the number of species that correctly did not pass the threshold. The TSS value was calculated by summing sensitivity (Eq. 4) and specificity (Eq. 5) subtracted by 1 (Eq. 6). As presented in Fig. 1d, the minimum occurrence threshold is the point at which the TSS value is initially maximized. In addition, the overall accuracy was calculated using Eq. 7. The indices, excluding the TSS value, range between 0 to 1, where 0 and 1 indicate totally incorrect and correct predictions, respectively. The TSS value ranges from –1 to 1, where –1 and 1 indicate totally incorrect and correct predictions, respectively, and 0 indicates that the prediction is random^47^. Although there is no specific classification for evaluating the TSS value, a value over 0.6 is generally considered a sufficiently acceptable result^42,43^.

4 $$\mathrm{Sensitivity }= \frac{a}{a+c}$$

5 $$\mathrm{Specificity }= \frac{d}{b+d}$$

6 $$\mathrm{TSS }=\mathrm{ Sensitivity }+\mathrm{ Specificity}-1$$

7 $$\mathrm{Overall\, accuracy }= \frac{a+d}{n}$$

All calculations were conducted using Microsoft Excel 2019 (Microsoft Corporation, Redmond, WA, USA).

## Data and materials availability

All data are available in the main text, supplementary materials, or from accessing the Water Environment Information System (https://water.nier.go.kr/). If website access is difficult, data can be obtained from the corresponding author on request.

### Supplementary Information

Supplementary Information.
