# Supplementary material for: A Benford’s law-based framework to determine the threshold of occurrence sites for species distribution modelling from ecological monitoring databases
Source: Sci Rep. 2023 Oct 5;13:16777. doi: 10.1038/s41598-023-44010-z (PMC10556063; doi:10.1038/s41598-023-44010-z)
Supplement: Supplementary file 1 — Supplementary Information. [file 41598_2023_44010_MOESM1_ESM.docx]

**Supplementary Data**

**A Benford’s Law-Based Framework to Determine the Threshold of Occurrence Sites for Species Distribution Modelling from Ecological Monitoring Databases**

Taeyong Shim^1^, Zhonghyun Kim^2^, Jisnho Jung^3,*^

^1^ Ojeong Resilience Institute, Korea University; Seoul 02841, Republic of Korea.

^2^ Institute of Life Science and Natural Resources, Korea University; Seoul 02841, Republic of Korea.

^3^ Division of Environmental Science & Ecological Engineering, Korea University; Seoul 02841, Republic of Korea.

^*^ Corresponding author. Email:

jjung@korea.ac.kr; Telephone: +82-2-3290-3066; Fax: +82-2-3290-3509

Table S1. Description of the freshwater fish monitoring dataset collected in this study. The collected data was sorted into three categories: “General” includes basic information of the fish monitoring program, “species” includes the information of fish species, and “observation” includes the results and statistics (total, average, minimum, and maximum) of the fish monitoring program.

| Category | Variables | Analysis^*^ |
| --- | --- | --- |
| General | Duration of monitoring program | W, S |
|  | Overall monitoring trials | W, S |
|  | Number of stations | W, S |
|  | Number of basins | W, S |
|  | Number of stations in each basin | W, S |
| Species | List of target species | W |
|  | Number of species observed | W |
|  | Number of species not observed | W |
|  | Number of common species | W |
|  | Number of alien species | W |
|  | Number of native species | W |
|  | Number of endangered species | W |
|  | Number of natural treasures | W |
| Observation | Overall catch | W |
|  | Average catch | W |
|  | Species’ total catch | W, S |
|  | Species’ average catch | W, S |
|  | Species’ maximum catch | W, S |
|  | Species’ minimum catch | W, S |
|  | Average of species’ total catch | W |
|  | Overall occurrence | W |
|  | Average of species’ occurrence | W |
|  | Species’ total occurrence | W, S |
|  | Number of occurred sites for each species | W, S |
|  | Number of occurred sites in each basin | W, S |

^*^ W and S indicate the whole and species-specific dataset, respectively, used to analyze the compliance with Benford’s law.

Table S2. Compliance of the freshwater fish dataset with Benford’s law, and the coefficient of determination (R^2^). Compliance was not evaluated (NE) for species with no occurrence records.

| Species | Number of occurred sites | R^2^ |
| --- | --- | --- |
| *Abbottina rivularis* | 244 | 0.990 |
| *Abbottina springeri* | 165 | 0.947 |
| *Acanthogobius flavimanus* | 42 | 0.926 |
| *Acanthogobius lactipes* | 28 | 0.869 |
| *Acanthorhodeus gracilis* | 379 | 0.969 |
| *Acanthorhodeus macropterus* | 299 | 0.983 |
| *Acentrogobius pflaumii* | 0 | NE |
| *Acheilognathus koreensis* | 350 | 0.973 |
| *Acheilognathus lanceolatus* | 706 | 0.991 |
| *Acheilognathus majusculus* | 56 | 0.970 |
| *Acheilognathus rhombeus* | 456 | 0.995 |
| *Acheilognathus signifer* | 107 | 0.969 |
| *Acheilognathus somjinensis* | 31 | 0.827 |
| *Acheilognathus yamatsutae* | 350 | 0.993 |
| *Anguilla japonica* | 48 | 0.861 |
| *Anguilla marmorata* | 0 | NE |
| *Aphyocypris chinensis* | 85 | 0.963 |
| *Aristichthys nobilis* | 0 | NE |
| *Boleophthalmus pectinirostris* | 3 | 0.433 |
| *Brachymystax lenok tsinlingensis* | 24 | 0.892 |
| *Carassius auratus* | 1967 | 0.980 |
| *Carassius cuvieri* | 236 | 0.985 |
| *Chaenogobius castaneus* | 19 | 0.856 |
| *Chaenogobius urotaenia* | 151 | 0.968 |
| *Channa argus* | 144 | 0.803 |
| *Chelon affinis* | 0 | NE |
| *Chelon haematocheilus* | 50 | 0.988 |
| *Cobitis hankugensis* | 301 | 0.966 |
| *Cobitis lutheri* | 311 | 0.940 |
| *Cobitis pacifica* | 21 | 0.754 |
| *Cobitis tetralineata* | 130 | 0.929 |
| *Coilia nasus* | 4 | 0.832 |
| *Coreoleuciscus splendidus* | 658 | 0.986 |
| *Coreoperca herzi* | 822 | 0.945 |
| *Coreoperca kawamebari* | 29 | 0.921 |
| *Cottus hangiongensis* | 10 | 0.840 |
| *Cottus koreanus* | 39 | 0.981 |
| *Ctenopharyngodon idellus* | 0 | NE |
| *Culter brevicauda* | 17 | 0.862 |
| *Cyprinus carpio* (Common carp) | 828 | 0.989 |
| *Cyprinus carpio* (Islaeli carp) | 16 | 0.965 |
| *Erythroculter erythropterus* | 223 | 0.989 |
| *Gasterosteus aculeatus* | 7 | 0.935 |
| *Gnathopogon strigatus* | 282 | 0.997 |
| *Gobiobotia brevibarba* | 79 | 0.912 |
| *Gobiobotia macrocephala* | 30 | 0.878 |
| *Gobiobotia nakdongensis* | 24 | 0.718 |
| *Hemibarbus labeo* | 833 | 0.978 |
| *Hemibarbus longirostris* | 846 | 0.979 |
| *Hemibarbus mylodon* | 67 | 0.992 |
| *Hemiculter eigenmanni* | 569 | 0.968 |
| *Hemiculter leucisculus* | 22 | 0.860 |
| *Hypomesus nipponensis* | 56 | 0.980 |
| *Hypophthalmichthys molitrix* | 0 | NE |
| *Hyporhamphus intermedius* | 6 | 0.577 |
| *Hyporhamphus sajori* | 12 | 0.851 |
| *Iksookimia choii* | 8 | 0.902 |
| *Iksookimia hugowolfeldi* | 62 | 0.979 |
| *Iksookimia koreensis* | 770 | 0.953 |
| *Iksookimia longicorpa* | 232 | 0.983 |
| *Iksookimia pumila* | 4 | 0.870 |
| *Iksookimia yongdokensis* | 33 | 0.966 |
| *Kichulchoia brevifasciata* | 2 | 0.106 |
| *Konosirus punctatus* | 15 | 0.894 |
| *Koreocobitis naktongensis* | 45 | 0.930 |
| *Koreocobitis rotundicaudata* | 322 | 0.946 |
| *Ladislabia taczanowskii* | 49 | 0.913 |
| *Lateolabrax japonicus* | 32 | 0.934 |
| *Lateolabrax maculatus* | 33 | 0.777 |
| *Lefua costata* | 43 | 0.939 |
| *Leiocassis nitidus* | 9 | 0.765 |
| *Leiocassis ussuriensis* | 90 | 0.934 |
| *Lepomis macrochirus* | 626 | 0.992 |
| *Lethenteron camtschaticum* | 1 | 0.434 |
| *Lethenteron reissneri* | 10 | 0.898 |
| *Liobagrus andersoni* | 233 | 0.986 |
| *Liobagrus mediadiposalis* | 227 | 0.989 |
| *Liobagrus obesus* | 7 | 0.779 |
| *Liobagrus somjinensis* | 62 | 0.906 |
| *Luciogobius guttatus* | 6 | 0.774 |
| *Macropodus ocellatus* | 88 | 0.956 |
| *Micropercops swinhonis* | 24 | 0.855 |
| *Microphysogobio jeoni* | 72 | 0.961 |
| *Microphysogobio koreensis* | 10 | 0.897 |
| *Microphysogobio longidorsalis* | 184 | 0.979 |
| *Microphysogobio rapidus* | 3 | 0.816 |
| *Microphysogobio yaluensis* | 920 | 0.984 |
| *Micropterus salmoides* | 1032 | 0.971 |
| *Misgurnus anguillicaudatus* | 1673 | 0.960 |
| *Misgurnus mizolepis* | 410 | 0.837 |
| *Monopterus albus* | 76 | 0.805 |
| *Mugil cephalus* | 114 | 0.995 |
| *Myoxocephalus stelleri* | 0 | NE |
| *Nipponocypris koreanus* | 1606 | 0.909 |
| *Niwaella multifasciata* | 166 | 0.986 |
| *Nuchequula nuchalis* | 9 | 0.612 |
| *Odontobutis interrupta* | 434 | 0.932 |
| *Odontobutis obscura* | 1 | 0.838 |
| *Odontobutis platycephala* | 1194 | 0.957 |
| *Oncorhynchus keta* | 6 | 0.739 |
| *Oncorhynchus masou masou* | 33 | 0.853 |
| *Oncorhynchus mykiss* | 9 | 0.906 |
| *Opsarichthys uncirostris amurensis* | 665 | 0.976 |
| *Oreochromis niloticus* | 9 | 0.964 |
| *Orthrias nudus* | 263 | 0.927 |
| *Orthrias toni* | 60 | 0.908 |
| *Oryzias latipes* | 142 | 0.974 |
| *Oryzias sinensis* | 230 | 0.981 |
| *Osmerus eperlanus mordax* | 2 | 0.274 |
| *Periophthalmus modestus* | 15 | 0.937 |
| *Periopthalmus magnuspinnatus* | 7 | 0.854 |
| *Phoxinus phoxinus* | 10 | 0.841 |
| *Platycephalus indicus* | 0 | NE |
| *Plecoglossus altivelis altivelis* | 187 | 0.980 |
| *Pseudobagrus brevicorpus* | 6 | 0.895 |
| *Pseudobagrus fulvidraco* | 343 | 0.924 |
| *Pseudobagrus koreanus* | 360 | 0.961 |
| *Pseudogobio esocinus* | 1523 | 0.969 |
| *Pseudopungtungia nigra* | 41 | 0.985 |
| *Pseudopungtungia tenuicorpa* | 98 | 0.980 |
| *Pseudorasbora parva* | 1207 | 0.985 |
| *Pungitius kaibarae* | 20 | 0.736 |
| *Pungitius sinensis* | 15 | 0.958 |
| *Pungtungia herzi* | 1711 | 0.979 |
| *Repomucenus olidus* | 5 | 0.755 |
| *Rhinogobius brunneus* | 1649 | 0.981 |
| *Rhinogobius giurinus* | 219 | 0.979 |
| *Rhodeus notatus* | 364 | 0.969 |
| *Rhodeus ocellatus* | 261 | 0.946 |
| *Rhodeus pseudosericeus* | 19 | 0.688 |
| *Rhodeus uyekii* | 621 | 0.976 |
| *Rhynchocypris kumgangensis* | 93 | 0.888 |
| *Rhynchocypris oxycephalus* | 1057 | 0.997 |
| *Rhynchocypris semotilus* | 0 | NE |
| *Rhynchocypris steindachneri* | 64 | 0.973 |
| *Sarcocheilichthys nigripinnis morii* | 314 | 0.989 |
| *Sarcocheilichthys variegatus wakiyae* | 253 | 0.987 |
| *Saurogobio dabryi* | 6 | 0.844 |
| *Silurus asotus* | 410 | 0.983 |
| *Silurus microdorsalis* | 130 | 0.958 |
| *siniperca scherzeri* | 170 | 0.899 |
| *Squalidus chankaensis tsuchigae* | 532 | 0.981 |
| *Squalidus gracilis majimae* | 1152 | 0.990 |
| *Squalidus japonicus coreanus* | 395 | 0.993 |
| *Squalidus multimaculatus* | 64 | 0.855 |
| *Squaliobarbus curriculus* | 85 | 0.976 |
| *Synechogobius hasta* | 36 | 0.932 |
| *Takifugu niphobles* | 15 | 0.933 |
| *Takifugu obscurus* | 1 | 0.179 |
| *Takifugu xanthopterus* | 0 | NE |
| *Trachidermus fasciatus* | 21 | 0.937 |
| *Tribolodon brandtii* | 2 | 0.713 |
| *Tribolodon hakonensis* | 52 | 0.960 |
| *Tridentiger bifasciatus* | 62 | 0.968 |
| *Tridentiger brevispinis* | 566 | 0.995 |
| *Tridentiger obscurus* | 73 | 0.981 |
| *Zacco platypus* | 2436 | 0.966 |
| *Zacco temminckii* | 457 | 0.976 |

Table S3. True skill statistics (TSS) to identify the minimum number (threshold) of fish occurrence sites. “a” is the number of species that comply with Benford’s law and pass the threshold; “b” is the number of species that do not comply with Benford’s law but pass the threshold; “c” is the number of species that comply with Benford’s law but fail to pass the threshold; “d” is the number of species that do not comply with Benford’s law and fail to pass the threshold.

|  |  | Compliance with Benford’s law | |
| --- | --- | --- | --- |
|  |  | Pass | Fail |
| Threshold (TSS) | Pass | a | b |
|  | Fail | c | d |
